# Dysregulation of C-X-C motif ligand 10 during aging and association with cognitive performance

**DOI:** 10.1016/j.neurobiolaging.2017.11.009

**Published:** 2018-03

**Authors:** Steven Bradburn, Jamie McPhee, Liam Bagley, Michael Carroll, Mark Slevin, Nasser Al-Shanti, Yoann Barnouin, Jean-Yves Hogrel, Mati Pääsuke, Helena Gapeyeva, Andrea Maier, Sarianna Sipilä, Marco Narici, Andrew Robinson, David Mann, Antony Payton, Neil Pendleton, Gillian Butler-Browne, Chris Murgatroyd

**Affiliations:** a School of Healthcare Science, Manchester Metropolitan University, Manchester, UK; b Institut de Myologie, UPMC UM 76, INSERM U 974, CNRS UMR, Paris, France; c Institute of Sport Sciences and Physiotherapy, Faculty of Medicine, University of Tartu, Tartu, Estonia; d Department of Human Movement Sciences, MOVE Research Institute, VU University Medical Center, Amsterdam, the Netherlands; e Department of Medicine and Aged Care, Royal Melbourne Hospital, University of Melbourne, Melbourne, Australia; f Gerontology Research Center, Faculty of Sport and Health Sciences, University of Jyväskylä, Jyväskylä, Finland; g Faculty of Medicine and Health Sciences, University of Nottingham, Derby, UK; h Division of Neuroscience and Experimental Psychology, School of Biological Sciences, The University of Manchester, Manchester, UK; i Centre for Epidemiology, Division of Population Health, Health Services Research & Primary Care, School of Health Sciences, The University of Manchester, Manchester, UK

**Keywords:** Cognitive aging, Alzheimer's disease, Inflammaging, DNA methylation, Epigenetics, Neurodegeneration

## Abstract

Chronic low-grade inflammation during aging (inflammaging) is associated with cognitive decline and neurodegeneration; however, the mechanisms underlying inflammaging are unclear. We studied a population (*n* = 361) of healthy young and old adults from the MyoAge cohort. Peripheral levels of C-X-C motif chemokine ligand 10 (CXCL10) was found to be higher in older adults, compared with young, and negatively associated with working memory performance. This coincided with an age-related reduction in blood DNA methylation at specific CpGs within the *CXCL10* gene promoter. In vitro analysis supported the role of DNA methylation in regulating *CXCL10* transcription. A polymorphism (rs56061981) that altered methylation at one of these CpG sites further associated with working memory performance in 2 independent aging cohorts. Studying prefrontal cortex samples, we found higher CXCL10 protein levels in those with Alzheimer's disease, compared with aged controls. These findings support the association of peripheral inflammation, as demonstrated by CXCL10, in aging and cognitive decline. We reveal age-related epigenetic and genetic factors which contribute to the dysregulation of *CXCL10*.

## 1 Introduction

Aging is associated with a heightened and prolonged systemic inflammation, termed inflammaging (Baylis et al., 2013, Franceschi and Campisi, 2014). The central nervous system (CNS) and peripheral immune system are inextricably linked and immunoregulatory signals support and shape the immune system (McAfoose and Baune, 2009, Schwartz et al., 2013). Accumulating evidence links chronic inflammation to cognitive decline and the risk of dementia. For example, mice lacking an adaptive immune system have reduced rates of neurogenesis (Ziv et al., 2006) and manifest cognitive deficits (Kipnis et al., 2004, Ziv et al., 2006). Furthermore, through the use of aging heterochronic parabiosis mouse models, excessive circulating proinflammatory cytokines in older organisms have been linked to neuronal insults and impaired spatial learning and memory (Villeda et al., 2011). In humans, older adults with high blood concentrations of proinflammatory cytokines perform worse on certain cognitive assessments (Baune et al., 2008, Trollor et al., 2012, Wilson et al., 2002) and are at an increased risk of dementia (Koyama et al., 2013), compared to those with low concentrations.

The molecular mechanisms underlying inflammaging, and the cognitive dysfunctions associated with it, are poorly understood. Since heterogeneity in the immune system between individuals are predominantly accountable by nonheritable influences (Brodin et al., 2015), the augmented inflammaging response may be a result of epigenetic disturbances. Epigenetics concerns mechanisms which mediate genetic control without altering the underling DNA sequence. DNA methylation, for example, is an epigenetic mediator of gene repression, and aging has been shown to dysregulate genome-wide DNA methylation marks (Jung and Pfeifer, 2015) and has been linked to neurodegeneration (Lardenoije et al., 2015).

Here, we investigated a panel of 35 plasma cytokines in physically and mentally healthy young and older human adults to identify age-related immune markers associated with specific measures of cognition. This revealed one cytokine, C-X-C motif chemokine ligand 10 (CXCL10), associated with spatial working memory. We further identified epigenetic mechanisms controlling age-related regulation of *CXCL10*, through DNA methylation at specific CpGs in the promoter. We then tested the relationship of a polymorphism, which removes one of these CpGs, with spatial working memory performance. Finally, we investigated CXCL10 within brain samples obtained from deceased individuals with pathological signs of intermediate Alzheimer's disease (AD) and compared them to aged controls.

## 2 Materials and methods

### 2.1 Study population

The study population analyzed was a part of the cross-sectional European multi-centre MyoAge cohort (McPhee et al., 2013). Those with full cognitive data and plasma samples available were included in this analysis (*n* = 361). Analysis consisted of young (*n* = 135) and relatively healthy older (*n* = 226) participants (98.9% Caucasian). A detailed description of the exclusion criteria, designed to ensure the selection of healthy participants and to minimize the confounding effect of comorbidity on sarcopenia, has been reported previously (McPhee et al., 2013). Information regarding lifestyle factors (such as education level, smoking status, and excessive alcohol intake) were self-reported. Excessive alcohol was defined for men as >21 units/week and for women >14 units/week. The local medical ethics committees of the respective institutions approved the study, and written informed consent was obtained from all participants.

### 2.2 Cognitive assessment

Participants completed the Mini–Mental State Examination (Folstein et al., 1975) and Geriatric Depression Scale (Yesavage et al., 1982) questionnaires to screen for cognitive impairment and depression, respectively. Exclusion criteria were set as; an Mini–Mental State Examination score of ≤23 and/or a Geriatric Depression Scale score of ≥5 points. Cognition (spatial working memory, executive functioning, and episodic memory) were assessed using the Cambridge Neuropsychological Test Automated Battery system (Cambridge Cognition Ltd). In addition, a global cognition score was determined as the sum of the 3 individual outcomes to represent a combined performance as utilized previously (Bradburn et al., 2016). Detailed information regarding each test and their output has been published previously (Bradburn et al., 2016). Each cognitive output was standardized by transforming into a *Z*-score based on the young group's average. A positive score therefore will indicate a higher than average performance and *vice versa*.

### 2.3 Plasma cytokine quantifications

Fasted plasma cytokines were quantified using either cytokine magnetic bead panels (MILLIPLEX MAP, Millipore; Supplementary Table 1) or sandwich ELISA assays (interleukin (IL) -1Ra, IL-6, IL-10, and tumor necrosis factor (TNF) -α; R&D Systems). For the multiplex immunoassays, assays included an overnight incubation at 4 °C and the use of a magnetic plate washer (Bio-Tek ELx405; Bio-Tek). Plates were processed on a Luminex 200 instrument (Luminex), and protein concentrations determined with the xPONENT software (Luminex, v. 3.1.871).

### 2.4 Plasma biochemical markers

Fasted plasma glucose, triglycerides, total cholesterol, high-density lipoprotein cholesterol and low-density lipoprotein cholesterol were all measured using a Daytona biochemical analyzer (Randox, County Antrim).

### 2.5 Blood cell counts

Lymphocyte and neutrophil counts were determined from whole blood using the KX-21 Automated Hematology Analyzer (Sysmex).

### 2.6 DNA extraction

Genomic DNA was extracted and purified from whole blood (buffy coat), cell cultures, and brain tissue samples using the Isolate II Blood DNA kit (Bioline), as per the manufacturer's instructions.

### 2.7 DNA bisulfite pyrosequencing

Genomic DNA (1 μg) was bisulfite treated via the EpiTect Fast Bisulfite Conversion kit (Qiagen). Primers were designed using the PyroMark Assay Design 2.0 (Qiagen) software and sequences are presented in Supplementary Table 2. The *CXCL10* proximal promoter region was amplified through polymerase chain reaction (PCR) using the PyroMark PCR reagents (Qiagen) with the following conditions: 95 °C (15 minutes), [94 °C (30 seconds), 56 °C (30 seconds), 72 °C (30 seconds)] (50 cycles), 72 °C (10 minutes). Bisulfite pyrosequencing was performed on the PyroMark Q24 system (Qiagen) as per the manufacturer's recommendations. A bisulfite conversion control was included in each sequencing assay to confirm complete bisulfite conversion of DNA.

### 2.8 Cell cultures

HeLa (ATCC) and U937 (ATCC) cells were maintained in Dulbecco Modified Eagle's medium and Roswell Park Memorial Institute medium 1640 media, respectively. Media were supplemented with 10% heat-inactivated FBS (Sigma), 2-mM L-glutamine (Lonza), and 200 U/mL Penicillin-Streptomycin (Lonza). Cells were maintained at 37 °C with 5% carbon dioxide.

### 2.9 Real-time polymerase chain reaction

Total RNA (2 μg) was extracted from U937 cells using TriSure (Bioline) and reverse transcribed into cDNA using the Tetro cDNA synthesis kit (Bioline) with the use of random hexamers. Primer sequences are found in Supplementary Table 2. Real-time polymerase chain reaction (RT-PCR) was performed on the Stratagene Mx3000P (Agilent) system using SYBR Green chemistry (SensiFAST HI-ROX, Bioline). RT-PCR was performed with the following program; 95 °C (2 minutes), [95 °C (5 seconds), 60 °C (10 seconds), 72 °C (10 seconds)] (40 cycles). All samples were analyzed in duplicate and the average cycle threshold value determined. Relative messenger RNA gene expression was calculated with the 2^−ΔΔCt^ equation (Livak and Schmittgen, 2001) using *GAPDH* and *ACTB* as internal controls.

### 2.10 *CXCL10* promoter plasmid and in vitro methylation

The pGL4-CXCL10 plasmid was used, which contains the full-length 972 bp human *CXCL10* promoter (−875 to +97 bp of the TSS), as created previously (Spurrell et al., 2005). The plasmid was subject to in vitro methylation treatment by using the CpG methyltransferase (*M.SssI*) enzyme (New England Biolabs), according to the manufacturer's instructions. For the unmethylated version, nuclease-free H_2_O was used instead of the *M.SssI* methylase enzyme. To methylate just the *CXCL10* promoter within the plasmid (patch methylation), the pGL4-CXCL10 plasmid (10 μg) was first double digested with *KpnI*-HF and *NheI*-HF (20 U each; New England Biolabs) restriction enzymes, and the products were gel extracted and purified via the NucleoSpin Gel and PCR Clean-up kit (Macherey-Nagel). The extracted *CXCL10* promoter insert (2 μg) was subject to the same aforementioned in vitro methylation treatment and purified with the NucleoSpin Gel and PCR Clean-up kit. The *CXCL10* promoter insert (200 ng) was ligated back into the pGL4 backbone using the Quick-Stick Ligase Kit (Bioline) using a vector-insert molar ratio of 1:4. Ligation efficiency was assessed by running on an agarose gel. Methylation of the CpG sites within the *CXCL10* proximal promoter for each construct was validated by bisulfite pyrosequencing.

### 2.11 Cell transfection and reporter assay

HeLa cells (0.5 × 10^4^/mL (500 μL)) were co-transfected with methylated or unmethylated pGL4-CXCL10 constructs and a pSV-β-Galactosidase control vector (Promega) using Lipofectamine 3000 (Thermo Fisher) for 24 hours. Reaction mixtures contained either 500 ng of whole pGL4-CXCL10 constructs or 20 μL of ligated patch methylation construct with 500 ng pSV-β-Galactosidase vector. Cells were washed twice with Dulbecco's phosphate-buffered saline before the addition of 10 ng/mL TNF-α and IFN-γ for 6 hours to stimulate *CXCL10* upregulation. Luciferase and β-galactosidase activities were simultaneously assessed from the same aliquot of cell lysate using the Dual-Light assay (Applied Biosystems). Luminescence was measured using the Synergy HT microplate reader (Bio-Tek) with automatic injectors used throughout. Assays were performed in duplicate and an average value calculated. Transfection normalization was performed by dividing the luciferase signal by the β-galactosidase signal to account for transfection efficiency. Results are presented as percentage activity relative to the unmethylated plasmids (100% activity).

### 2.12 Genotyping

In total, 3 single nucleotide polymorphisms (SNPs) were genotyped: rs56061981 (*CXCL10*), rs7412 (*APOE*), and rs429358 (*APOE*). The rs56061981 and rs7412 SNPs were simultaneously detected using iPLEX technology by CIGMR Biobank (The University of Manchester, UK), a fee-for-service provider, using the Sequenom system. The rs429358 SNP was analyzed using a TaqMan assay (Cat# 4351379; Thermo Fischer Scientific). The rs429358 and rs7412 genotypes were used to determine the *APOE* ε4 allele frequency (Belbin et al., 2007). The genotyping completion rate for the SNPs was: 96.4% rs7412 (13 failures), 98.9% rs429358 (4 failures), and 97.8% rs56061981 (8 failures).

### 2.13 Replicative genetic cohort

The University of Manchester Age and Cognitive Performance Research Cohort (ACPRC), and the linked Dyne-Steele DNA archive for cognitive genetics, were used as a replicate population for the genetic analysis of the rs56061981 polymorphism with a similar spatial working memory score as in the MyoAge cohort. Recruitment methodology for the ACPRC has been described in detail elsewhere (Rabbitt et al., 2004). Only those which contained data on: rs56061981 genotype, spatial working memory performance, age at testing and gender were included in this analysis (*n* = 1493; age = 65.26 ± 6.16 years; 71.1% female). Spatial working memory performance from the ACPRC was measured by the memory circle test, as described previously (Rabbitt et al., 2004). Briefly, participants were shown a 12-quadrant circle containing a line drawing of recognizable objects and asked to immediately recall the names and the location of the objects. The number of object names and their corresponding location successfully recalled is herein classed as the spatial working memory score (12 maximum).

### 2.14 Prefrontal cortex samples

Fresh, frozen human prefrontal cortex samples (*n* = 67) were acquired from the Manchester Brain Bank of participants from the ACPRC (Supplementary Table 3). Ethical approval was granted from the Manchester Brain Bank Committee. Confirmation of pathological diagnosis was determined by a trained pathologist and with the use of immunohistochemical techniques. Where applicable, samples were stratified into 2 groups termed aged (Braak stage: 0–II; CERAD: ≤ A) and intermediate AD (Braak stage: III–IV; CERAD: B; Thal stage: ≥ 1) (Supplementary Table 4). Samples which contained potentially confounding pathologies, such as those related to Parkinson's disease, were excluded from subgrouping.

### 2.15 CXCL10 protein brain lysate quantification

Approximately 100 mg of tissue was used for CXCL10 protein quantification. Briefly, tissues were lysed with RIPA buffer (Sigma) supplemented with 1× protease inhibitor cocktail (Sigma) and 0.1-M PMSF (Sigma). Total protein quantification from lysates was determined using the Pierce BCA Protein Assay Kit (Thermo Scientific). Quantification of lysate CXCL10 protein was determined with the Human CXCL10/IP-10 DuoSet ELISA (R&D Systems). CXCL10 levels were normalized relative to the total protein in the assay (pg/mg of total protein).

### 2.16 Statistical analysis

All statistical analyses were performed using IBM SPSS Statistics 23 and the level of significance was set at *p* < 0.05, unless otherwise stated. For population characteristics, normally distributed continuous data were analyzed by independent Student *t* tests and presented as mean ± standard deviation. Otherwise, Mann-Whitney U tests were performed and results were presented as median and interquartile range. Categorical data were analyzed via Pearson χ^2^. Cytokine protein concentrations were natural log (ln) transformed before analysis and compared using Mann-Whitney U tests.

Based on a posteriori evidence, those cytokines which differed between age groups were analyzed further through Spearman partial correlations (1-tailed analysis) to control for gender, participant location, and various confounding variables. A confounding variable was defined if any of the anthropometry, lifestyle, or cardiovascular health markers listing in Table 1 had a relationship with the cytokines through a Spearman correlation at the *p* < 0.1 level (2-tailed analysis), as used previously (Baune et al., 2008, Bradburn et al., 2016, Wright et al., 2006). Owing to multiple testing effects (17 tests), a Bonferroni corrected significance threshold of *p* < 0.003 (0.05/17) was also applied here.

**Table 1:** Characteristics of the MyoAge cohort used within this analysis

Variable	Young	Old	*p* value
Age, y	23.42 (2.74)	74.47 (3.38)	<0.001
Females, *n* (%)	71 (52.6)	116 (51.3)	0.816 (*X*^2^, 1 = 0.05)
Location, *n* (%)			
Holland	33 (24.4)	71 (31.4)	0.223 (*X*^2^, 4 = 5.70)
Finland	34 (25.2)	68 (30.1)	
Estonia	33 (24.4)	47 (20.8)	
France	17 (12.6)	17 (7.5)	
UK	18 (13.3)	23 (10.2)	
Anthropometry			
Height, m	1.74 (0.09)	1.68 (0.09)	<0.001
Body mass, kg	68.95 (11.40)	71.45 (12.32)	0.055
BMI, kg/m^2^	22.71 (2.76)	25.36 (3.35)	<0.001
Lifestyle			
Current smoker, *n* (%)	14 (10.4)	11 (4.9)	0.046 (*X*^2^, 1 = 3.97)
Excessive alcohol use, *n* (%)	15 (11.1)	19 (8.4)	0.395 (*X*^2^, 1 = 0.72)
Cardiovascular health			
Glucose, mmol/L	4.89 (0.57)	5.38 (0.77)	<0.001
Triglyceride, mmol/L	0.92 (0.44)	1.14 (0.49)	<0.001
Total cholesterol, mmol/L	4.37 (0.92)	5.36 (0.97)	<0.001
HDL cholesterol, mmol/L	1.49 (0.40)	1.65 (0.40)	<0.001
LDL cholesterol, mmol/L	2.50 (0.80)	3.18 (0.87)	<0.001
Education^a^			
Basic school, *n* (%)	0 (0)	33 (17.8)	<0.001 (*X*^2^, 2 = 66.91)
High school, *n* (%)	14 (12.3)	70 (37.8)	
University, *n* (%)	100 (87.7)	82 (44.3)	
Mental state			
MMSE score (points), median (IQR)^b^	30 (29–30)	29 (28–30)	<0.001
GDS score (points), median (IQR)^c^	0 (0–1)	1 (0–2)	0.001
APOE genotype^d^			
One or more *APOE* ε4 alleles present, *n* (%)	40 (30.1)	46 (21.3)	0.065 (*X*^2^, 1 = 3.42)

All of the data presented are the mean (standard deviation), unless otherwise stated.

Key: GDS, Geriatric Depression Scale; HDL, high-density lipoprotein; LDL, low-density lipoprotein; MMSE, Mini–Mental State Examination.

a Data available in *n* = 299 (young, *n* = 114; old, *n* = 185).

b Data available in *n* = 357 (young, *n* = 134; old, *n* = 223).

c Data available in *n* = 316 (young, *n* = 116; old, *n* = 200).

d Data available in *n* = 349 (young, *n* = 133; old, *n* = 216).

To determine the relationship between age-related cytokines and measures of cognitive performance in the older adults, Spearman correlations (2-tailed analysis) were first applied. Significant relationships were analyzed further through Spearman partial correlations (1-tailed analysis) to determine if the relationship between the cytokine concentration and cognition remained after controlling for gender, participant location, age, education level, and *APOE* ε4 presence. Owing to multiple testing effects (3 tests), a Bonferroni corrected significance threshold of *p* < 0.017 (0.05/3) was applied.

Age differences for DNA methylation at CpG sites between young and old adults were performed using independent Student *t* tests. Correlations between CpG site methylation levels were determined with Pearson's correlation tests. Age correlations with DNA methylation, while controlling for white blood cell populations (neutrophil and lymphocyte percentages), were performed using Spearman partial correlations.

Comparisons between control and stimulated cell culture models were determined with independent Student *t* tests. Differences in *CXCL10* promoter activity was calculated with Mann-Whitney U tests.

Relationships between the rs56061981 polymorphism and spatial working memory scores were assessed through separate linear regression models (additive and dominant) using the PLINK software (v.1.90b3.36). Models included adjustments for age at testing and gender (and participant location for the MyoAge cohort). A positive standardized coefficient (β) indicates the minor allele increases the spatial working memory performance mean and *vice versa*.

Correlations between *CXCL10* DNA methylation and protein in prefrontal cortex samples with age were analyzed with Spearman correlation. Differences in methylation between genotypes and protein levels between controls and those with intermediate AD neuropathologies were conducted with Mann-Whitney U tests.

## 3 Results

To investigate age-related interactions between inflammation and aging, we studied a cohort of physiologically and cognitively healthy individuals as part of the MyoAge study (McPhee et al., 2013). These were age stratified into 2 groups, young (range: 18–30 years) and old (range: 69–81 years). Their characteristics are presented in Table 1.

### 3.1 Age-related differences in cytokines between healthy young and old

**Fig. 1:**
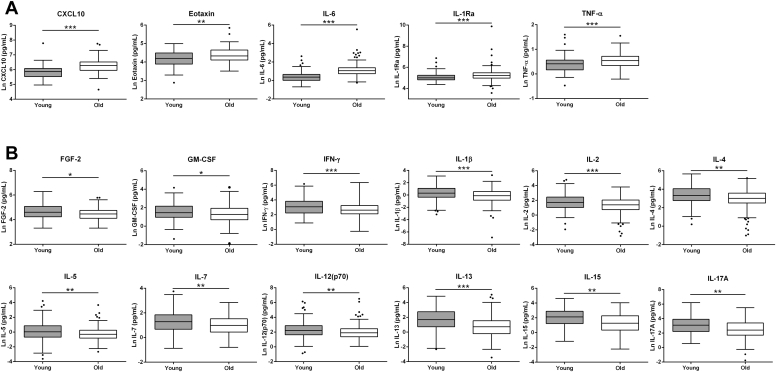
Plasma cytokines which were significantly different between the young and old adult group. (A) Plasma cytokines which were significantly higher in the older adult group compared to the younger adult group. (B) Plasma cytokines which were significantly lower in the older adult group compared to the younger adult group. Young *n* = 44–135; old *n* = 64–225. ^∗^*p* < 0.05; ^∗∗^*p* < 0.01; ^∗∗∗^*p* < 0.001.

Plasma concentrations of 35 inflammatory cytokines were measured in the young and old groups. Seventeen (49%) of the cytokines measured were significantly different between the age groups (Fig. 1). However, when controlling for gender, participant location, and various predetermined confounding variables through nonparametric partial correlation analysis, only CXCL10, eotaxin-1, and IL-6 remained significantly associated with participant ages (Supplementary Table 5).

### 3.2 Relationship between age-related cytokines and measures of cognitive performance in the older adults

To determine whether any cytokines might associate with cognition and age, those cytokines which were significantly associated with age were tested using Spearman correlation with the 4 cognitive performance scores in the older adults. Supplementary Table 6 displays the correlation coefficients for each relationship. CXCL10 was negatively associated with spatial working memory, and eotaxin-1 was negatively associated with episodic memory and global cognition scores.

To further account for confounders, we next used nonparametric partial correlation analysis to control for participant age, gender, location, education level, and *APOE* ε4 allele presence. Only the relationship between CXCL10 and spatial working memory remained after adjustments (Table 2). This association remained when BMI was also introduced as a confounder (*rho*_*adjusted*_ = −0.237, *p* = 0.014).

**Table 2:** Partial correlation between age-related plasma cytokines and cognition after controlling for multiple confounders in the older adults

Relationship	Adjusted correlation coefficient (*rho*)	*p* value
CXCL10 and spatial working memory	−0.250	0.010^a^
Eotaxin-1 and episodic memory	−0.168	0.060
Eotaxin-1 and global cognition	−0.174	0.054

Controlling for: age, gender, location, education, and *APOE* ε4 allele presence.

One-tailed analysis; *n* = 85.

Key: CXCL10, C-X-C motif chemokine ligand 10.

a Signifies that the correlation is significant after accounting for multiple testing via the Bonferroni correction method (*p* < 0.017).

### 3.3 DNA methylation at the *CXCL10* proximal promoter in aging and its association with plasma protein levels

**Fig. 2:**
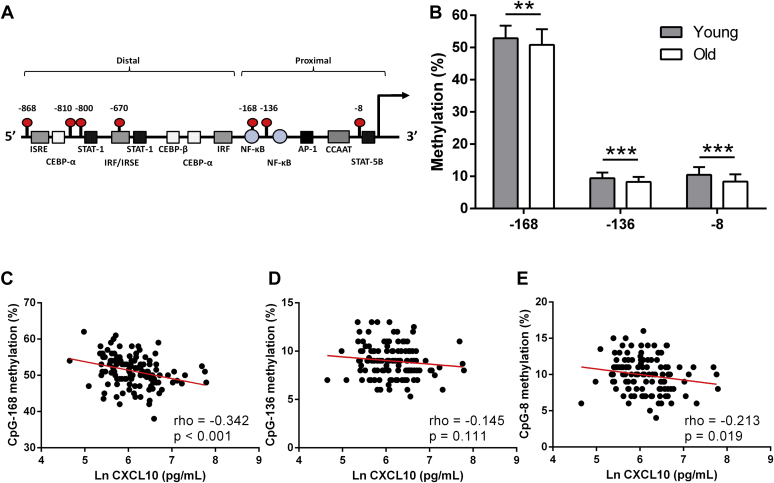
DNA methylation differences between age groups for the CXCL10 proximal promoter and associations with plasma protein concentrations. (A) Full-length human *CXCL10* promoter region (negative strand shown). Transcription factor binding sites were predicted using MatInspector (core: >0.9; matrix similarity: optimized). CpG sites are represented as a red beacon with the positions relative to the transcriptional start site (extended arrow) in base pairs. There are 4 and 3 CpG sites located in the distal and proximal regions respectively. (B) Differences in *CXCL10* proximal promoter methylation between young and old adults. Young *n* = 69; old *n* = 82. ^∗∗^*p* < 0.01; ^∗∗∗^*p* < 0.001. Association between plasma CXCL10 levels (ln transformed) with CpG-168 (C), CpG-136 (D), and CpG-8 (E) methylation levels. *n* = 122. Abbreviations: AP-1, activating protein 1; CCAAT, CAT box; CEBP-α, CCAAT/enhancer binding protein alpha; CEBP-β, CCAAT/enhancer binding protein beta; IRF, interferon regulatory factor; CXCL10, C-X-C motif chemokine ligand 10; ISRE, IFN-stimulatory element; NF-κB, nuclear factor-kappa B; STAT, signal transducers and activators of transcription. (For interpretation of the references to color in this figure legend, the reader is referred to the Web version of this article.)

We next investigated whether epigenetic changes in DNA methylation occur at the *CXCL10* gene with age, and whether this might relate to the age-associated regulation of plasma CXCL10 levels. In a random subset of participants, DNA methylation of the 3 CpG sites within the proximal *CXCL10* promoter (Fig. 2A) were quantified from blood samples via bisulfite pyrosequencing. DNA methylation was significantly lower in the older adults in comparison to the younger adults at all 3 CpG sites investigated (CpG-168, CpG-136 and CpG-8) (Fig. 2B).

Relationships between each CpG site were investigated to determine if they are regulated together. There was a significant positive association between methylation at sites closest in proximity, that is, CpG-168 and CpG-136 (*r* = 0.381, *p* < 0.001) as well as CpG-136 and CpG-8 (*r* = 0.624, *p* < 0.001).

To test whether levels of CpG methylation might critically relate to cell heterogeneity within the samples, we next investigated the relationship between average CpG methylation at the proximal *CXCL10* promoter with age while controlling for white blood cell population changes (neutrophil and lymphocyte percentages). Analysis revealed a negative correlation between participant age and average CpG methylation (*rho* = −0.368, *p* < 0.001) which remained after accounting for neutrophil and lymphocyte frequencies in the blood (*rho*_adjusted_ = −0.212, *p* = 0.032).

Spearman correlation analysis was also used to deduce the possible relationships between *CXCL10* promoter methylation and CXCL10 plasma protein levels. A significant negative association between plasma CXCL10 protein with CpG-168 (Fig. 2C) and CpG-8 (Fig. 2E) was found. No association was found with CpG-136 (Fig. 2D). After controlling for participant age, only the relationship between CpG-168 methylation with CXCL10 plasma protein levels remained (*rho*_adjusted_ = −0.255, *p* = 0.002).

### 3.4 DNA methylation and regulation of *CXCL10* promoter activity

**Fig. 3:**
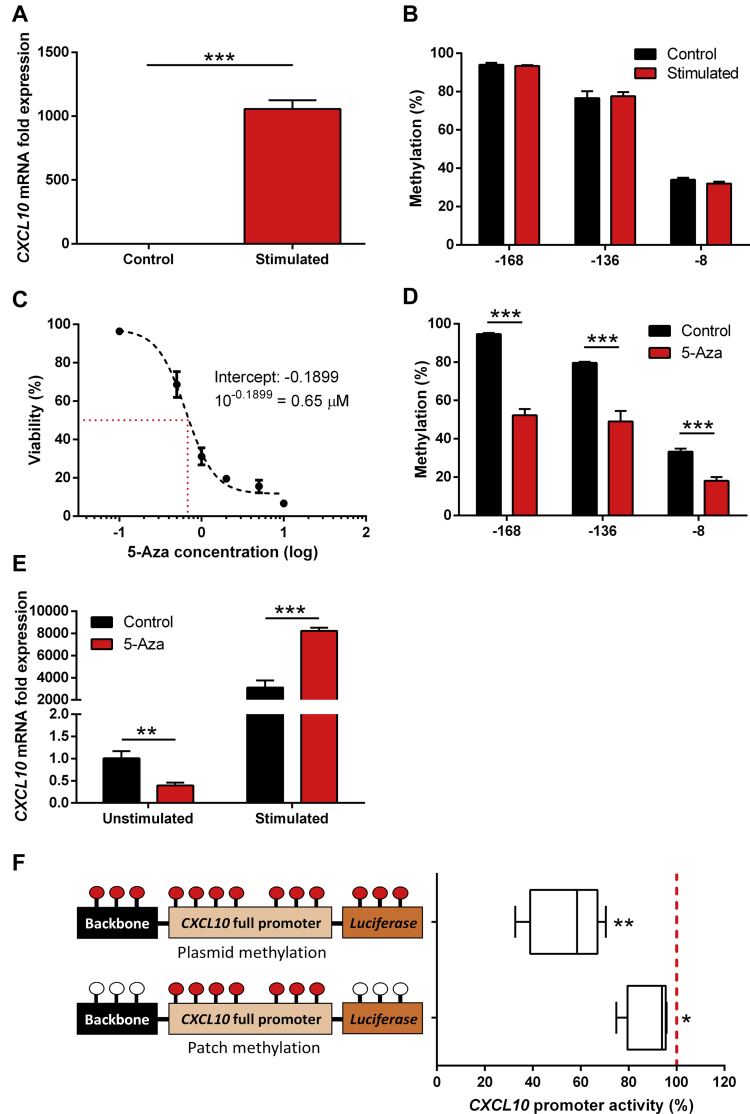
Analysis of CXCL10 regulation, DNA methylation, and promoter activity. (A) CXCL10 mRNA expression in U937 cells following stimulation with 10 ng/mL TNF-α and IFN-γ for 90 minutes. Values are normalized to GAPDH and BACTIN expression. *n* = 3. (B) DNA methylation from U937 cells for the 3 CpG sites following stimulation with TNF-α and IFN-γ. *n* = 3. (C) The half inhibitory concentration of U937 cells exposed to 5-azacytidine (5-Aza). Cell viability was determined by the trypan blue exclusion method. Briefly, U937 cells were seeded at 1 × 10^5^ in a 24-well plate overnight. The next day cells were incubated with various concentrations (0.1, 0.2, 1, 2, 5, and 10 μM) of 5-Aza for 72 hours. Fresh 5-Aza was added every 24 hours. Red line indicates a cell viability of 50%. *n* = 3. (D) DNA methylation from U937 cells treated with and without 5-Aza for the 3 CpG sites. Cells were incubated with 0.65 μM 5-Aza for 72 hours, with fresh 5-Aza added every 24 hours, before stimulation. *n* = 3. (E) CXCL10 mRNA expression in U937 cells pre-treated with 5-Aza before and after the addition of TNF-α and IFN-γ. Values are normalized to GAPDH and BACTIN expression. Simulated levels of CXCL10 mRNA gene expression are relative to their unstimulated counterparts. *n* = 3. (A–E) ^∗∗^*p* < 0.01; ^∗∗∗^*p* < 0.001. (F) Promoter activity of the CXCL10 promoter following in vitro methylation. Schematics denote either plasmid methylation or patch methylation techniques. Beacons represent methylated (red) or unmethylated (white) CpG's. Luciferase values were normalized via the β-galactosidase signal to account for transfection efficiency, and results are expressed as the promoter percentage activity relative to the unmethylated constructs (100% activity; red dotted line). *n* = 4–5. ^∗^*p* < 0.05; ^∗∗^*p* < 0.01 relative to corresponding unmethylated constructs. Abbreviations: CXCL10, C-X-C motif chemokine ligand 10; mRNA, messenger RNA; TNF, tumor necrosis factor. (For interpretation of the references to color in this figure legend, the reader is referred to the Web version of this article.)

To investigate whether DNA methylation at the *CXCL10* promoter regulates activity, we used in vitro systems. *CXCL10* transcription is activated in monocytes through synergistic induction by TNF-α and interferon (IFN) -γ (Qi et al., 2009). Simulating U937 cells (a human monocyte-like cell line) with TNF-α and IFN-γ resulted in a 1000-fold upregulation of *CXCL10* messenger RNA (Fig. 3A). However, this did not alter DNA methylation at the *CXCL10* proximal promoter (Fig. 3B).

We then tested the effects of DNA hypomethylation on *CXCL10* promoter activity by incubating U937 cells with the DNA methyltransferase inhibitor 5-azacytidine (5-Aza) for 72 hours. Prior optimization determined that 0.65-μM 5-Aza was the half inhibitory concentration (IC_50_) of cell viability in U937 cells; therefore this concentration was used during the experiment (Fig. 3C). 5-Aza treatment induced a significant reduction in DNA methylation at each of the CpG sites within the *CXCL10* proximal promoter region (Fig. 3D). Testing *CXCL10* gene activity, we found that before cell stimulation, those incubated with 5-Aza had a twofold reduction in *CXCL10* gene expression (Fig. 3E). Following the stimulation with TNF-α and IFN-γ, those cells pre-incubated with 5-Aza produced a twofold augmented *CXCL10* gene induction when compared with those without 5-Aza (Fig. 3E).

To understand the effect of DNA hypermethylation on *CXCL10* promoter activity, we used *M.SssI* methyltransferase to methylate a plasmid construct containing the full human *CXCL10* promoter region and transfected these into the HeLa cells. Methylation in vitro was conducted on either the entire pGL4-CXCL10 plasmid or just the *CXCL10* full promoter region, which contains 7 CpGs in total (Fig. 2A), through a patch methylation technique. Whole plasmid methylation resulted in a 45% reduction of *CXCL10* promoter activity, whereas methylating just the *CXCL10* promoter insert resulted in a 10% reduction in promoter activity (Fig. 3F).

### 3.5 Relationship between rs56061981 polymorphism, DNA methylation, and spatial working memory performance

**Fig. 4:**
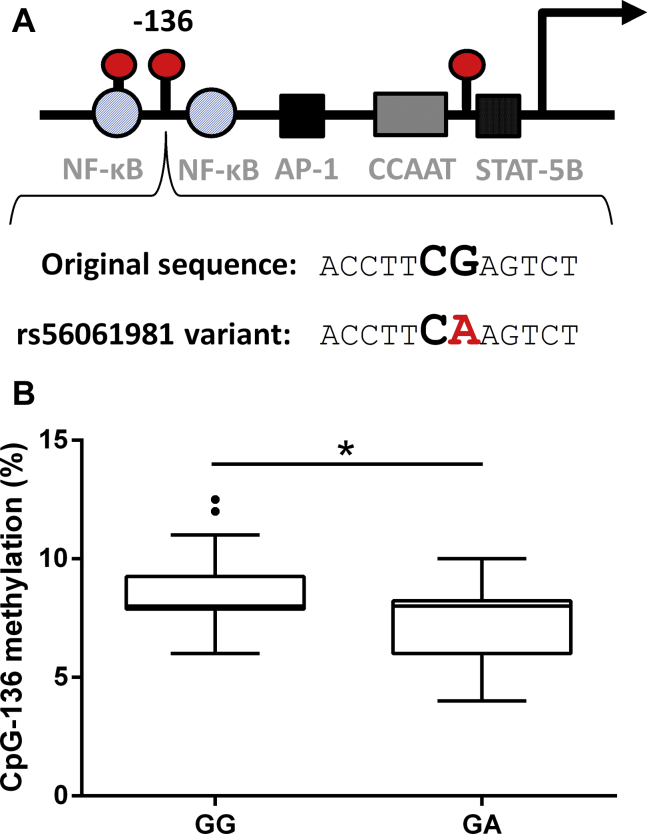
DNA methylation differences between rs56061981 genotypes in the healthy young and old adults. (A) Schematic representation of CXCL10 proximal promoter showing the rs56061981 polymorphism and the resulting change in the CpG-136 site (negative strand shown). (B) Difference between CpG-136 methylation in GG (*n* = 65) and GA (*n* = 14) carriers in the old group. ^∗^*p* < 0.05. Abbreviation: CXCL10, C-X-C motif chemokine ligand 10.

We next investigated whether a polymorphism (rs56061981), that effectively removes the CpG-136 site through a guanine (G) to adenine (A) substitution (Fig. 4A), could influence *CXCL10* promoter methylation and in turn relate to cognition. Specifically, we focussed on spatial working memory since CXCL10 protein levels were significantly associated with this particular cognitive measure.

Methylation at the CpG-136 site was significantly lower in the heterozygotes (GA) when compared to the wild-type homozygotes (GG) in the old adults (Fig. 4B). AA homozygotes were not included in the analysis as there was only one in our study population. There was no difference in plasma CXCL10 levels (*p* = 0.730) and methylation at the CpG-168 (*p* = 0.787) or CpG-8 (*p* = 0.210) between the GG and GA genotypes.

We next tested if the rs56061981 polymorphism was related to spatial working memory performance in the old adults by using a linear regression analysis to adjust for participant age at testing, gender, and subject location. This demonstrated a significant positive association between the rs56061981 genotype and spatial working memory scores in the additive and dominant genetic models in the older adult group (Table 3). The low frequency of AA homozygotes (*n* = 1) did not allow to test recessive genetic models. To substantiate this finding, we tested for an association between rs56061981 genotype with a spatial working memory test from ACPRC (Rabbitt et al., 2004). This replicative cohort again revealed a similar significant association between the rs56061981 genotype and spatial working memory performance (Table 3).

**Table 3:** Relationship between rs56061981 polymorphism and spatial working memory performance in old adults

Cohort/genetic effect	β	95% CI		*p* value
		Lower	Upper	
MyoAge^a^				
Additive	0.13	0.00	0.26	0.050
Dominant	0.17	0.04	0.30	0.009
ACPRC^b^				
Additive	0.09	0.04	0.14	<0.001
Dominant	0.09	0.04	0.14	<0.001

Confounders adjusted for: age at testing and gender (and participant location for the MyoAge cohort).

Key: β, standardized beta coefficient; ACPRC, The University of Manchester Age and Cognitive Performance Research Cohort.

a The rs56061981 SNP was deemed to be within the Hardy-Weinberg equilibrium (frequency: 201 (GG), 19 (GA), 1 (AA); *p* = 0.393).

b The rs56061981 SNP was deemed to be within the Hardy-Weinberg equilibrium (frequency: 1385 (GG), 107 (GA), 1 (AA); *p* = 0.719).

### 3.6 *CXCL10* promoter methylation and protein levels within prefrontal cortex

**Fig. 5:**
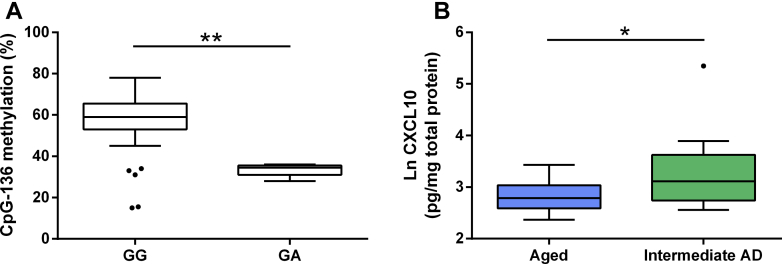
Comparison of CXCL10 methylation and protein levels in the prefrontal cortex. (A) Difference between CpG-136 methylation in GG (*n* = 61) and GA (*n* = 5) carriers in the prefrontal cortex samples. (B) CXCL10 protein levels from the prefrontal cortex samples of those showing intermediate pathological signs of AD and those with no neuropathologies. Aged *n* = 17; intermediate AD *n* = 16. ^∗^*p* < 0.05; ^∗∗^*p* < 0.01. Abbreviations: AD, Alzheimer's disease; CXCL10, C-X-C motif chemokine ligand 10.

To explore mechanisms underlying *CXCL10* regulation in the aging brain, we tested *CXCL10* promoter methylation and protein levels in brains, specifically prefrontal cortex samples from the ACPRC. There was no correlation between *CXCL10* proximal promoter methylation (CpG-168: *rho* = −0.136, *p* = 0.274; CpG-136: *rho* = −0.059, *p* = 0.637; CpG-8: *rho* = 0.154, *p* = 0.212) or CXCL10 protein levels (*rho* = −0.187, *p* = 0.132) and age at death of the subjects. Methylation at the CpG-136 site was significantly lower in the heterozygotes (GA) when compared with the wild-type homozygotes (GG; Fig. 5A).

Next, based on neuropathological data, we stratified samples into 2 groups: intermediate AD and aged controls. There was no difference in DNA methylation at the *CXCL10* proximal promoter between the 2 groups (CpG-168: *p* = 0.260; CpG-136: *p* = 0.382; CpG-8: *p* = 1.000). Those classified as having intermediate AD had significantly higher CXCL10 protein levels in the prefrontal cortex compared with the aged controls (Fig. 5B).

## 4 Discussion

The results of this study further support the role of inflammation in cognitive aging. We found higher concentrations of numerous proinflammatory cytokines in old, compared with young adults. The cytokine CXCL10 was significantly associated with a measure of cognitive performance, specifically spatial working memory, in the older adults. Examining regulatory mechanisms, we further detected age-associated reductions of DNA methylation at CpG sites within the *CXCL10* proximal promoter region in the blood. DNA methylation at specific sites within this region was negatively correlated with CXCL10 plasma concentrations. Using in vitro systems, we showed that DNA methylation within the *CXCL10* promoter represses expression. Further, an SNP within the promoter region which removes a CpG site and reduces methylation, was found to positively associate with spatial working memory in 2 independent cohorts of older adults. Finally, investigating the possible role of CXCL10 regulation in the brain, we found higher CXCL10 protein levels in prefrontal cortex samples of those showing intermediate pathological signs of AD compared with aged controls.

Initially, we described higher plasma CXCL10, eotaxin-1, and IL-6 in the older adults, even when accounting for confounding factors. These age-related associations are in agreement with findings from previous aging cohorts (Antonelli et al., 2006, Stowe et al., 2010, Villeda et al., 2011). Of these, only CXCL10 associated with cognitive performance when accounting for confounders in the older adults. Interestingly, plasma CXCL10 levels have been linked to cognitive status in Parkinson's disease (Rocha et al., 2014) and are elevated in the cerebrospinal fluid of mild cognitive impaired patients (Galimberti et al., 2006). Investigations using neuronal cell cultures have suggested that excess CXCL10 can act as a neurotoxic agent by promoting apoptosis, as demonstrated in human fetal neuronal cell cultures (Sui et al., 2006), human cholinergic neurons (van Marle et al., 2004), human brain vascular endothelial cells (Wilson et al., 2013), and neuroglia (Wilson et al., 2013). Therefore, from a pathological perspective, excessive exposure of the CNS to CXCL10 may be detrimental for neuronal functioning and cognitive performance.

Next, we found that DNA methylation at the *CXCL10* proximal promoter region was significantly lower in the blood of older adults when compared with the younger adults. This age-related hypomethylation is also apparent in other inflammatory genes, such as *TNF-α* (Gowers et al., 2011) and *IL-1β* (Cho et al., 2015), thus further suggesting a contribution of DNA methylation loss in inflammaging. Since buffy coat samples contain a heterogeneous cell population, therefore potentially influencing DNA methylation results (Reinius et al., 2012), we further confirm that the age-related hypomethylation is independent of the differences in the variabilities of certain blood cell types (neutrophils and lymphocytes). *CXCL10* hypomethylation with age is also in agreement with a previous methylation array study in aging monozygotic twins, specifically a reduction of methylation at the CpG-168 site (ID: cg14622819) with age was found (Fernández et al., 2015). In addition, the CpG-800 site (ID: cg23884076) within the distal promoter region (not analyzed here) was also hypomethylated during aging on the same array (Fernández et al., 2015). Collectively there seems to be an age-related loss of DNA methylation within the *CXCL10* promoter from the blood.

DNA methylation is a known gene repressor; though RNA analysis could not be tested on the blood samples, we did find a negative association between DNA methylation at CpG-168 of the *CXCL10* proximal promoter with plasma proteins levels suggesting some level of control over gene regulation. Interestingly, CpG-168 is located within a nuclear factor-kappa B (NF-κB) binding site. NF-κB is a transcriptional regulator involved in numerous pathways related to inflammation, including cytokine production and apoptosis (Hayden and Ghosh, 2012) and is a potent activator of *CXCL10* (Yeruva et al., 2008). Furthermore, DNA methylation within these binding sites has been known to inhibit NF-κB binding (Bednarik et al., 1991). Complementing this potential *CXCL10* gene regulation involving DNA methylation, through the use of a DNA methyltransferase inhibitor (5-Aza), we observed a significant hypomethylation of the proximal promoter coinciding with an aberrant *CXCL10* upregulation following stimulation of cells with TNF-α and IFN-γ. These results are supportive to those of Peng et al. who incubated primary ovarian cancer cells with 5-aza-2′-deoxycytidine, a deoxy derivative of 5-Aza, which resulted in the hypomethylation of an STAT-1 binding site upstream of the promoter region and an exacerbated release of *CXCL10* following IFN-γ stimulation (Peng et al., 2015). STAT-1 is required for IFN-γ–induced CXCL10 production in monocytes (Qi et al., 2009), and STAT-1 binding affinity has been shown to be directly inhibited following DNA methylation (Chen et al., 2000). On the other hand, by hypermethylating the *CXCL10* promoter region in vitro, we further show a 10% reduction in promoter activity therefore suggesting that DNA methylation directly contributes to *CXCL10* gene inhibition. It is worth noting that methylating the whole plasmid resulted in further promoter repression, most likely due to the methylation of CpG sites within the backbone and luciferase gene. Collectively, these results suggest that *CXCL10* promoter hypomethylation may prime regulatory regions thereby augmenting gene induction when stimulated. Furthermore, since DNA methylation of the full promoter only explained 10% of the promoter activity, it is unlikely to be the sole contributor to *CXCL10* regulation. Other epigenetic meditators, such as histone modifications (Burke et al., 2013, Coward et al., 2010, Peng et al., 2015) and chromatin remodeling (Hudy et al., 2013), have also been shown to contribute to the transcriptional control of *CXCL10* and therefore may account for the additional variance in promoter activity.

Importantly, the described inhibitory effects of DNA methylation on the *CXCL10* regulation are all based on stimulated systems, rather than basal conditions. Surprisingly, despite the hypomethylation of the proximal promoter region, we found a twofold reduction in *CXCL10* gene expression in unstimulated U937 cells following 5-Aza treatment. Here, activating transcription factors (e.g., NF-κB p65 and STAT-1) on the *CXCL10* promoter should not be present, therefore, it may be possible, therefore, that repressive transcription factors are further downregulating *CXCL10*. For example, an NF-κB repressing factor binding element is present within the *CXCL10* promoter and NF-κB repressing factor has been shown to directly inhibit basal *CXCL10* expression in monocytes (Huang et al., 2013, Huang et al., 2014).

CpG-136 investigated here forms part of the polymorphism rs56061981. This SNP has previously been shown to alter the binding affinity of nuclear proteins and augments *CXCL10* expression and protein production (Deng et al., 2008). Also, these specific types of SNPs associated with CpG sites have been shown to directly influence local DNA methylation (Hellman and Chess, 2010). Based on this evidence, we tested for differences in DNA methylation at this site between genotypes and found a lower methylation of CpG-136 in the heterozygotes in the blood (Fig. 4B) and brain (Fig. 5A).

We next investigated the relationship between the rs56061981 genotype and cognitive performance in the older adults. Since we previously found a link between spatial working memory and CXCL10 protein levels, we limited our investigations to this cognitive measure in older adults. There was a positive association between spatial working memory performance and the rs56061981 genotype. This relationship was also apparent in a separate larger aging cohort. Overall, these associations suggest a better spatial working memory performance with the presence of the minor allele, conferring polymorphic hypomethylation. Considering previous reports suggest an increase in gene activity in the rs56061981 carriers (Deng et al., 2008, Singh et al., 2017), the association of better spatial working memory performance in those affected was unexpected. However, it is worth noting that the prior functional evaluation of the rs56061981 genotype on *CXCL10* activity was based on IFN-γ stimulated, rather than basal, peripheral mononuclear cells (Deng et al., 2008). Further work is therefore needed to elaborate on the association between rs56061981, methylation, and spatial working memory performance in older adults particularly at the cellular level.

By using prefrontal cortex samples from ACPRC (Rabbitt et al., 2004), we were able to provide an insight into the regulation of *CXCL10* within the human brain. We failed to find any relationship between CXCL10 protein or methylation levels with the age at death of the participants. This lack of association may be accounted for by the smaller age range of participants investigated (range: 72–104 years old). A larger age range, particularly including samples from younger individuals, may be required to confirm possible age-related CXCL10 changes within the brain. We next investigated differences in between a group with intermediate pathological AD and nonpathological aged controls. The intermediate AD group had significantly higher levels of CXCL10 protein compared with the aged, however, *n* differences in DNA methylation were observed. This would suggest the increase in CXCL10 protein within the brain during AD pathogenesis is independent of DNA methylation regulation. It is worth mentioning, however, that our results are limited to brain tissues, as opposed to cell-specific analysis, which may have an influence on the methylation findings. The increase in CXCL10 protein is in agreement with reports describing increased CXCL10 staining, especially within astrocytes, in close proximity to amyloid-β deposits (Xia et al., 2000). There is also evidence of an increase in cerebral *Cxcl10* gene expression (Krauthausen et al., 2015) and protein levels, most notably by microglia (Krauthausen et al., 2015) and astrocytes (Lai et al., 2013), in rodent AD models. Interestingly, cerebrospinal fluid levels of CXCL10 have been suggested to peak at the time of clinical mild cognitive impairment to AD conversion in humans (Brosseron et al., 2014). The associations between CXCL10 protein within the CNS and periphery during early AD pathogenesis warrant the need for further investigations around this important disease stage.

## 5 Conclusions

In summary, we showed that the inflammatory profile is significantly altered by aging. The proinflammatory age-related cytokine, CXCL10, was negatively correlated with spatial working memory in community-dwelling older adults. The age-associated upregulation of plasma CXCL10 coincided with a reduction in leukocyte DNA methylation within the gene promoter region. Loss of DNA methylation within this region resulted in an increased *CXCL10* promoter activity. An SNP shown to induce hypomethylation correlated with a better spatial working memory performance in 2 separate populations of older adults. Finally, higher protein levels were also observed in the prefrontal cortex of those with intermediate pathological AD. This study suggests that age-related polymorphic and epigenetic dysregulations of key inflammatory genes, such as *CXCL10*, may be involved in inflammaging and neurodegeneration.

## Disclosure statement

The authors have no conflicts of interest to disclose.
