# Supplementary material for: Dysregulation of C-X-C motif ligand 10 during aging and association with cognitive performance
Source: Neurobiol Aging. 2018 Mar;63:54–64. doi: 10.1016/j.neurobiolaging.2017.11.009 (PMC5805841; doi:10.1016/j.neurobiolaging.2017.11.009)
Supplement: Supplementary Tables 1–6 [file mmc1.docx]

**Supplementary material**

**Supplementary Table 1. Assay sensitivity for the cytokines measured using multiplex immunoassays**

| Protein | Abbreviation | Sensitivity (pg/mL) |
| --- | --- | --- |
| *Cat# HCYTMAG-60K* |  |  |
| C-X-C motif chemokine 10 | CXCL10 | 8.6 |
| Epidermal growth factor | EGF | 2.8 |
| Eotaxin-1 | Eotaxin-1 | 4.0 |
| Fibroblast growth factor 2 | FGF-2 | 7.6 |
| Fms-related tyrosine kinase 3 ligand | FLT-3L | 5.4 |
| Fractalkine | Fractalkine | 22.7 |
| Granulocyte-colony stimulating factor | G-CSF | 1.8 |
| Growth regulated oncogene | GRO | 9.9 |
| Interferon alpha 2 | IFN-α2 | 2.9 |
| Interleukin 1 alpha | IL-1α | 9.4 |
| Interleukin 3 | IL-3 | 0.7 |
| Interleukin 9 | IL-9 | 1.2 |
| Interleukin 12 p40 | IL-12 (p40) | 7.4 |
| Interleukin 15 | IL-15 | 1.2 |
| Interleukin 17 A | IL-17A | 0.7 |
| Monocyte chemoattractant protein 1 | MCP-1 | 1.9 |
| Macrophage-derived chemokine | MDC | 3.6 |
| Macrophage inflammatory protein 1 alpha | MIP-1α | 2.9 |
| Soluble CD40 ligand | sCD40L | 5.1 |
| Transforming growth factor alpha | TGF-α | 0.8 |
| Tumour necrosis factor beta | TNF-β | 1.5 |
| *Cat# HSCYTO-60SPMX* |  |  |
| Granulocyte macrophage colony-stimulating factor | GM-CSF | 0.35 |
| Interferon gamma | IFN-γ | 0.48 |
| Interleukin 1 beta | IL-1β | 0.14 |
| Interleukin 2 | IL-2 | 0.19 |
| Interleukin 4 | IL-4 | 1.12 |
| Interleukin 5 | IL-5 | 0.12 |
| Interleukin 7 | IL-7 | 0.42 |
| Interleukin 8 | IL-8 | 0.13 |
| Interleukin 12 p70 | IL-12 (p70) | 0.15 |
| Interleukin 13 | IL-13 | 0.23 |

**Supplementary Table 2. Primer sequences and efficiencies for RT-PCR and bisulfite pyrosequencing**

| Gene | Primer | Application | Sequence (5’ – 3’) | Efficiency (%) |
| --- | --- | --- | --- | --- |
| *CXCL10* | Forward | RT-PCR | TCCACGTGTTGAGATCATTGC | 103 |
|  | Reverse |  | TCTTGATGGCCTTCGATTCTG |  |
| *GAPDH* | Forward | RT-PCR | CCGCATCTTCTTTTGCGTCG | 91 |
|  | Reverse |  | TGGAATTTGCCATGGGTGGA |  |
| *BACTIN* | Forward | RT-PCR | CATCCTCACCCTGAAGTACC | 92 |
|  | Reverse |  | ATAGCAACGTACATGGCTGG |  |
| *CXCL10* | Forward | Bisulfite pyrosequencing | AAGAGGAGTAGAGGGAAATT | N/A |
|  | Reverse |  | BIO-AATTCCTCTACTATAAACTCAAAATATATC |  |
|  | Sequencing1 |  | GGAGTAGAGGGAAATTT |  |
|  | Sequencing2 |  | GGTTTTGTAGATAAATATGGTATA |  |

Key: *BIO*, biotin tag; *N/A*, not applicable.

**Supplementary Table 3. Characteristics of the participants used for prefrontal cortex analysis**

|  | *n* = 67 |
| --- | --- |
| Age at death, years | 87.51 (6.07) |
| Females, *n* (%) | 46 (68.7) |
| Whole brain weight, g^A^ | 1207.37 (137.37) |
| Post-mortem delay, hours^B^ | 76.14 (43.68) |
| *APOE* ε4 allele presence, *n* (%) | 22 (32.8) |

^A^ *n* = 43. ^B^ *n* = 60. All of the data presented is mean (standard deviation), unless otherwise stated.

**Supplementary Table 4. Characteristics of the prefrontal cortex samples stratified by pathological diagnosis**

|  | Aged  (*n* = 17) | Intermediate AD  (*n* = 16) | *p* value |
| --- | --- | --- | --- |
| Age at death, years | 87.47 (5.35) | 87.69 (6.92) | 0.920 |
| Females, *n* (%) | 12 (70.6) | 12 (75.0) | 1.00^C^ |
| Whole brain weight, g^A^ | 1147.70 (117.68) | 1213.00 (134.94) | 0.245 |
| Post-mortem delay, hours^B^ | 74.44 (48.61) | 77.69 (35.95) | 0.838 |
| *APOE*ε4 allele presence, *n* (%) | 3 (17.6) | 6 (37.5) | 0.259^C^ |
| Thal stage (amyloid deposition), *n* (%) |  |  |  |
| None | 8 (47.1) | 0 | <0.001^C^ (*X*^2^, 5 = 21.533) |
| 1 | 5 (29.4) | 0 |  |
| 2 | 2 (11.8) | 3 (18.8) |  |
| 3 | 2 (11.8) | 7 (43.8) |  |
| 4 | 0 | 3 (18.8) |  |
| 5 | 0 | 3 (18.8) |  |
| CERAD score (neuritic plaques), *n* (%) |  |  |  |
| None | 9 (52.9) | 0 | <0.001^C^ (*X*^2^, 2 = 37.301) |
| A (Sparse) | 8 (47.1) | 0 |  |
| B (Moderate) | 0 | 16 (100.0) |  |
| C (Frequent) | 0 | 0 |  |
| Braak stage (neurofibrillary tangles), *n* (%) |  |  |  |
| None | 3 (17.6) | 0 | <0.001^C^ (*X*^2^, 2 = 37.840) |
| I – II | 14 (82.4) | 0 |  |
| III – IV | 0 | 16 (100.00) |  |
| V – VI | 0 | 0 |  |

^A^ Aged: *n* = 10; intermediate AD: *n* = 12. ^B^ Aged: *n* = 16; intermediate AD: *n* = 18. ^C^ Fisher’s Exact Test. All of the data presented is mean (standard deviation), unless otherwise stated. AD = Alzheimer’s disease.

**Supplementary Table 5. Partial correlation between age-related plasma cytokines and age after controlling for gender, participant location and various confounders**

| Cytokine | *n* | Adjusted correlation coefficient (*rho*) | *p* value | Variables controlled for; gender, location and: |
| --- | --- | --- | --- | --- |
| CXCL10 | 174 | 0.217 | 0.002† | Height; BMI; smoking status; glucose; triglycerides; total cholesterol |
| Eotaxin-1 | 179 | 0.235 | 0.001† | Body mass; BMI |
| FGF-2 | 156 | -0.008 | 0.459 | BMI; smoking status; glucose; triglycerides; total cholesterol; LDL cholesterol; |
| GM-CSF | 352 | -0.040 | 0.228 | Excessive alcohol intake |
| IFN-γ | 350 | -0.082 | 0.062 | Excessive alcohol intake; total cholesterol; LDL cholesterol |
| IL-1β | 345 | -0.083 | 0.061 | Excessive alcohol intake; total cholesterol; LDL cholesterol; |
| IL-1Ra | 346 | 0.101 | 0.030 | Height; body mass; BMI; glucose; triglycerides; total cholesterol; HDL cholesterol; LDL cholesterol |
| IL-2 | 348 | -0.097 | 0.035 | Height; excessive alcohol intake; total cholesterol; HDL cholesterol |
| IL-4 | 322 | -0.036 | 0.262 | BMI; excessive alcohol intake; total cholesterol; LDL cholesterol |
| IL-5 | 346 | -0.064 | 0.118 | Excessive alcohol intake; total cholesterol |
| IL-6 | 348 | 0.381 | <0.001† | Height; body mass; BMI; glucose; triglycerides; total cholesterol; LDL cholesterol |
| IL-7 | 353 | -0.082 | 0.062 | Excessive alcohol intake |
| IL-12(p70) | 349 | -0.062 | 0.124 | Excessive alcohol intake; triglycerides; total cholesterol; LDL cholesterol |
| IL-13 | 283 | -0.113 | 0.028 | BMI; glucose; triglycerides; total cholesterol; LDL cholesterol |
| IL-15 | 100 | -0.165 | 0.049 | Total cholesterol; HDL cholesterol; LDL cholesterol |
| IL-17A | 171 | 0.080 | 0.147 | BMI; glucose; triglycerides; total cholesterol; LDL cholesterol |
| TNF-α | 144 | 0.144 | 0.004 | Body mass; BMI; excessive alcohol use; glucose; triglycerides; LDL cholesterol |

† Signifies the correlation is significant after accounting for multiple testing via the Bonferroni correction method (*p* < 0.003). 1-tailed analysis.

**Supplementary Table 6. Relationship between age-related plasma cytokines and cognition in the older adults**

|  | Spatial working memory | Executive functioning | Episodic memory | Global cognition |
| --- | --- | --- | --- | --- |
| CXCL10 | -0.258** | 0.079 | -0.004 | -0.032 |
| Eotaxin-1 | -0.038 | -0.118 | -0.201* | -0.229* |
| IL-6 | -0.002 | 0.019 | 0.102 | 0.067 |

Results presented are Spearman correlation coefficient (*rho*). * *p* < 0.05; ** *p* < 0.01. 2-tailed analysis.
